# Privacy-Preserving Generation of Structured Lymphoma Progression Reports from Cross-sectional Imaging: A Comparative Analysis of Llama 3.3 and Llama 4

**DOI:** 10.1007/s10278-025-01618-z

**Published:** 2025-07-25

**Authors:** Philipp Prucker, Keno K. Bressem, Su Hwan Kim, Dominik Weller, Avan Kader, Felix J. Dorfner, Sebastian Ziegelmayer, Markus M. Graf, Tristan Lemke, Florian Gassert, Elif Can, Aymen Meddeb, Daniel Truhn, Martin Hadamitzky, Marcus R. Makowski, Lisa C. Adams, Felix Busch

**Affiliations:** 1 Institute for Diagnostic and Interventional Radiology, School of Medicine and Health, TUM University Hospital rechts der Isar, Technical University of Munich, Munich, Germany; 2 Institute of Diagnostic and Interventional Neuroradiology, School of Medicine and Health, TUM University Hospital rechts der Isar, Technical University of Munich, Munich, Germany; 3 Institute for Cardiovascular Radiology and Nuclear Medicine, School of Medicine and Health, TUM University Hospital German Heart Center, Technical University of Munich, Munich, Germany; 4 Department of Radiology, Charité – Universitätsmedizin Berlin, Corporate Member of Freie Universität Berlin and Humboldt Universität zu Berlin, Berlin, Germany; 5 Department of Interventional Radiology, Faculty of Medicine, Medical Center - University of Freiburg, Freiburg im Breisgau, Germany; 6 Department of Neuroradiology, Charité – Universitätsmedizin Berlin, Corporate member of Freie Universität Berlin and Humboldt-Universität zu Berlin, Berlin, Germany; 7 Department of Neuroradiology, Hôpital Maison-Blanche, CHU Reims, Université Reims-Champagne-Ardenne, Reims, France; 8 Berlin Institute of Health at Charité, Universitätsmedizin Berlin, Berlin, Germany; 9 Department of Radiology, University Hospital RWTH Aachen, Aachen, Germany

**Keywords:** Lymphoma, Artificial intelligence, Medical informatics, Natural language processing, Radiology, Large language models

## Abstract

Efficient processing of radiology reports for monitoring disease progression is crucial in oncology. Although large language models (LLMs) show promise in extracting structured information from medical reports, privacy concerns limit their clinical implementation. This study evaluates the feasibility and accuracy of two of the most recent Llama models for generating structured lymphoma progression reports from cross-sectional imaging data in a privacy-preserving, real-world clinical setting. This single-center, retrospective study included adult lymphoma patients who underwent cross-sectional imaging and treatment between July 2023 and July 2024. We established a chain-of-thought prompting strategy to leverage the locally deployed Llama-3.3-70B-Instruct and Llama-4-Scout-17B-16E-Instruct models to generate lymphoma disease progression reports across three iterations. Two radiologists independently scored nodal and extranodal involvement, as well as Lugano staging and treatment response classifications. For each LLM and task, we calculated the F1 score, accuracy, recall, precision, and specificity per label, as well as the case-weighted average with 95% confidence intervals (CIs). Both LLMs correctly implemented the template structure for all 65 patients included in this study. Llama-4-Scout-17B-16E-Instruct demonstrated significantly greater accuracy in extracting nodal and extranodal involvement information (nodal: 0.99 [95% CI = 0.98–0.99] vs. 0.97 [95% CI = 0.95–0.96], *p* < 0.001; extranodal: 0.99 [95% CI = 0.99–1.00] vs. 0.99 [95% CI = 0.98–0.99], *p* = 0.013). This difference was more pronounced when predicting Lugano stage and treatment response (stage: 0.85 [95% CI = 0.79–0.89] vs. 0.60 [95% CI = 0.53–0.67], *p* < 0.001; treatment response: 0.88 [95% CI = 0.83–0.92] vs. 0.65 [95% CI = 0.58–0.71], *p* < 0.001). Neither model produced hallucinations of newly involved nodal or extranodal sites. The highest relative error rates were found when interpreting the level of disease after treatment. In conclusion, privacy-preserving LLMs can effectively extract clinical information from lymphoma imaging reports. While they excel at data extraction, they are limited in their ability to generate new clinical inferences from the extracted information. Our findings suggest their potential utility in streamlining documentation and highlight areas requiring optimization before clinical implementation.

**Supplementary Information:**

The online version contains supplementary material available at 10.1007/s10278-025-01618-z.

## Introduction

Accurate tumor staging and disease surveillance are critical for effective treatment planning and monitoring in oncology patients. In an era of precision oncology, characterized by evolving personalized treatment regimens and prolonged disease courses that demand regular staging examinations, frequent cross-sectional imaging has become indispensable [1, 2]. However, a major challenge is the processing and analysis of free-text radiology reports, which can vary considerably between interpreting radiologists [3]. This challenge is exacerbated by the increasing number of imaging studies, which underscores the need for efficient data processing, particularly for the aggregated presentation of disease progression in multidisciplinary tumor boards, for which numerous reports need to be reviewed, condensed, and synthesized to support informed interdisciplinary treatment planning [4, 5].

Large language models (LLMs) have demonstrated remarkable capabilities in extracting structured information from unstructured radiology reports, enabling standardized and interpretable outputs [6]. Their application in radiology could streamline documentation processes, enhance workflow efficiency, and improve clinical decision-making by analyzing and processing vast amounts of clinical data [7]. In addition, LLMs have shown promise in automating tumor staging by extracting relevant information and applying established classification systems such as the TNM staging [8].

However, the integration of LLMs in healthcare is hindered by concerns regarding data privacy, security, and regulatory compliance. Proprietary models, such as OpenAI’s Generative Pre-Trained Transformers (GPT), do not disclose their underlying algorithms, training data, or data processing and storage mechanisms, thereby hindering accessibility, optimization for the medical domain, the assessment of potential biases, reproducibility, and compliance with local medical standards and regulations [9]. In addition, many popular proprietary models rely on external cloud-based processing, which requires data transmission outside of secure hospital environments, posing serious risks to patient confidentiality and fail to comply with privacy regulations such as the Health Insurance Portability and Accountability Act (HIPAA) and the General Data Protection Regulation (GDPR) [10, 11]. Consequently, a key requirement for the safe and effective implementation of LLMs in clinical practice is local deployment within secure hospital infrastructures, enabling compliance with privacy policies while preserving the benefits of streamlining data management [12]. Yet, while the feasibility of proprietary LLMs for extracting structured data from imaging reports has been explored, hardly any study has implemented and evaluated LLMs in a secure environment using genuine patient data in real-world clinical settings [13–21].

Therefore, this study aims to evaluate the feasibility and clinical accuracy of open-source LLMs, specifically two of the most recent Llama 3.3 and 4 models, in a local, privacy-preserving hospital setting, to generate structured summary reports including lymphoma disease involvement, Lugano staging, and Lugano treatment response classification from cross-sectional imaging reports. By operating within a hospital’s secure computing environment, this approach aims to mitigate data security risks while assessing whether the model can extract and organize radiological data and generate clinically relevant conclusions that align with expert decision-making.

## Methods

This retrospective, single-center study evaluated the use of Llama-3.3-70B-Instruct and Llama-4-Scout-17B-16E-Instruct in a local, privacy-preserving hospital setting to generate structured lymphoma progression reports based on cross-sectional radiology reports collected between July 2023 and July 2024. The study was approved by the institutional review board of Technical University of Munich (2024–590-S-CB). Informed consent was waived as the data collection was part of routine clinical care.

### Inclusion and Exclusion Criteria

This study included adult patients (≥ 18 years) with a pathologically confirmed diagnosis of lymphoma who received cross-sectional imaging at Technical University of Munich between July 2023 and July 2024. Patients were eligible if they had at least two complete CT studies performed at our institution, along with corresponding German-language radiology reports, and underwent treatment within the examination interval. Patients with non-lymphoma malignancies or those under 18 years of age were excluded from the analysis.

### Local LLM Infrastructure

Both models were deployed on a dedicated hospital server running an Ollama server (https://github.com/ollama/ollama) that hosts Llama-3.3-70B-Instruct and Llama-4-Scout-17B-16E-Instruct behind the institutional firewall [22, 23]. The system is secured to allow access only to authorized users, ensuring that only personnel with approved credentials can interact with the model via open-webui (https://github.com/open-webui/open-webui). All application programming interface calls, with the temperature parameter set to zero, and web-based communications are over encrypted channels (Hypertext Transfer Protocol Secure) within the hospital network, preventing any data from leaving the secure environment. This configuration meets institutional privacy requirements by restricting data processing to on-premises resources, thereby preserving patient confidentiality throughout the restructuring and processing of radiology report text.

### Prompt Design

**Fig. 1:**
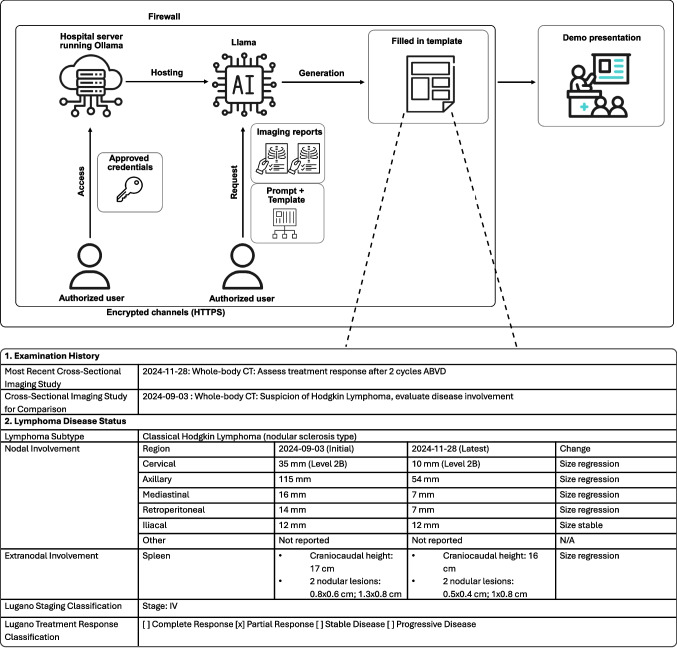
Schematic illustration of a privacy-preserving, locally deployed large language model (LLM) workflow for report generation in a hospital setting. Interaction with the LLM takes place entirely within a hospital’s cloud infrastructure, protected by a firewall. Patient data security is maintained through multiple layers: perimeter security (firewall), access control (authorized users), encryption (secret key), and local model deployment. The locally hosted Llama models generate a structured disease progression report from the provided input (template, cross-sectional imaging reports), as illustrated below with a representative fictional case. This architecture ensures compliance with health data security frameworks, such as the Health Insurance Portability and Accountability Act, by processing protected health information exclusively within the institution’s secure environment, eliminating external data transmission risks. Abbreviations: HTTPS, Hypertext Transfer Protocol Secure; N/A, not applicable

We employed a chain-of-thought (CoT) prompting strategy to generate structured disease progression reports from cross-sectional imaging results. First, the LLM was prompted to extract key data from radiology reports, including lymphoma subtype, exam details, and measurements of nodal and extranodal sites. The LLM then chronologically compared these findings across time points to identify changes. Subsequently, using the criteria provided in the prompt, the model was asked to determine the Lugano stage and Lugano treatment response based on the extracted data from the previous tasks [24–26]. Finally, the model was prompted to fill in all information into a structured template. As a reference for the reader, we have provided all prompts used in the supplements (see Supplementary Material, 1. Prompting strategy). Figure 1 illustrates a schematic workflow of the local, privacy-friendly LLM setting used in our study, including an example of a completed template.

### Evaluation Criteria

The evaluation framework consisted of five criteria. First, we evaluated the correctness of extracted information at the [1] nodal and [2] extranodal levels (i.e., sites identified, size measurements correct, and longitudinal changes correctly stated). Second, we validated the correctness of the LLM-generated [3] Lugano stage and [4] treatment response classifications. Since these classifications were not explicitly stated in the source reports, this step aimed to assess the models’ ability to apply the clinical rules provided in the prompt to the previously extracted findings. Lugano staging modifiers A and B were excluded from the analysis as the B-symptoms were not necessarily detailed in the radiology reports. Finally, we verified that [5] the output correctly adhered to the specified template structure provided in the prompt. Two residents in radiology with 1 and 5 years of experience in cross-sectional imaging, respectively, independently evaluated each generated report by comparing them against the original radiology reports and assessing the Lugano staging/treatment response criteria. After initial evaluation, inter-rater agreement was assessed, and any discrepancies were resolved through consensus with a senior radiologist with 10 years of experience in cross-sectional imaging. All tasks were evaluated at the exam level across three iterations.

### Statistical Analysis

All statistical analyses were conducted using Statistical Package for the Social Sciences (version 30.0.0, International Business Machines Corporation, Armonk, NY, USA). The Shapiro–Wilk test was used to assess normality, and as all variables were non-normally distributed, numerical data were reported as medians with interquartile ranges (IQR), while categorical variables were summarized as frequencies and percentages. Inter-rater agreement was calculated using Cohen’s *κ* for nodal/extranodal involvement and quadratic-weighted *κ* for both Lugano staging and treatment response classification, including 95% confidence intervals (95% CI). For each task, we calculated F1, accuracy, recall, precision, and specificity per label as well as the case-weighted average with 95% CIs. Two-sided paired McNemar *χ*^2^ test with continuity correction on the discordant counts was used for model comparison. Statistical significance was defined as a two-sided* p*-value < 0.05.

## Results

### Patient Characteristics

Sixty-five adult lymphoma patients underwent cross-sectional imaging at our institution between July 2023 and July 2024 and were included for analysis. Median age was 46 years (IQR = 31–62), and 50.8% of patients were female. The most common lymphoma subtypes were diffuse large B-cell lymphoma (27.7%), Hodgkin lymphoma (21.5%), and follicular lymphoma (21.5%). Most patients (24.6%) had Stage III [2] according to the Ann Arbor classification, followed by Stage I (20.0%), and Stage IV (18.5%). More than half of the patients (52.3%) had mediastinal lymph node involvement, while splenic lesions were the most common extranodal site, affecting 21.5% of patients. Detailed patient basic characteristics are presented in Table 1.

**Table 1:** Basic characteristics of the study cohort

Variable	Value (*N* = 65)
Gender, *n* (%)	
Male	32 (49.2)
Female	33 (50.8)
Age, years	
Median (IQR)	46 (31–62)
Lymphoma type, *n* (%)	
DLBCL	18 (27.7)
Hodgkin lymphoma	14 (21.5)
Follicular lymphoma	14 (21.5)
MALT lymphoma	6 (9.2)
Marginal zone lymphoma	5 (7.7)
T-cell lymphoma	4 (6.2)
Mantle cell lymphoma	3 (4.6)
Burkitt lymphoma	1 (1.5)
Lugano staging classification, *n* (%)	
Stage I	13 (20.0)
Stage IE	8 (12.5)
Stage II	7 (10.8)
Stage IIE	8 (12.5)
Stage III(1)	1 (1.5)
Stage III(2)	16 (24.6)
Stage IV	12 (18.5)
Nodal location, *n* (%)	
Mediastinal lymph nodes	34 (52.3)
Cervical lymph nodes	21 (32.3)
Retroperitoneal lymph nodes	19 (29.2)
Axillary lymph nodes	13 (20.0)
Iliac lymph nodes	13 (20.0)
Inguinal/femoral lymph nodes	8 (12.5)
Mesenteric lymph nodes	4 (6.2)
Thoracic nodes (extra-mediastinal)	3 (4.6)
Supraclavicular lymph nodes	2 (3.1)
Generalized lymphadenopathy	1 (1.5)
Infraclavicular lymph nodes	1 (1.5)
Bilateral hilar lymph nodes	1 (1.5)
Unilateral hilar lymph nodes	1 (1.5)
Extranodal location, *n* (%)	
Splenic lesions	14 (21.5)
Pulmonary lesions	12 (18.5)
Hepatic lesions	4 (6.2)
Epicardial involvement	2 (3.1)
Bone marrow involvement	3 (4.6)
Gastric infiltration	1 (1.5)
Thyroid involvement	1 (1.5)
Cutaneous involvement	1 (1.5)
Lugano response classification, *n* (%)	
Complete response	12 (18.5)
Partial response	36 (55.4)
Stable disease	10 (15.4)
Progressive disease	7 (10.8)
Imaging reports per patient, median (IQR)	
CT	4 (3–5)
PET-CT	2 (2–3)
*DLBCL* diffuse large B-cell lymphoma, *IQR* interquartile range, *MALT* mucosa-assisted lymphoid tissue	

### Inter-rater Agreement

Inter-rater reliability between the two radiology residents was excellent, with Cohen’s *κ* = 0.91 (95% CI = 0.90–0.93) for nodal involvement and 0.93 (95% CI = 0.91–0.95) for extranodal involvement. Quadratic-weighted *κ* was 0.90 (95% CI = 0.83–0.95) for Lugano staging and 0.90 (95% CI = 0.83–0.95) for Lugano treatment response assessments.

### Performance Analysis

Both models produced complete and syntactically correct templates in all 195 cases.

#### Performance for Nodal/Extranodal Involvement

**Fig. 2:**
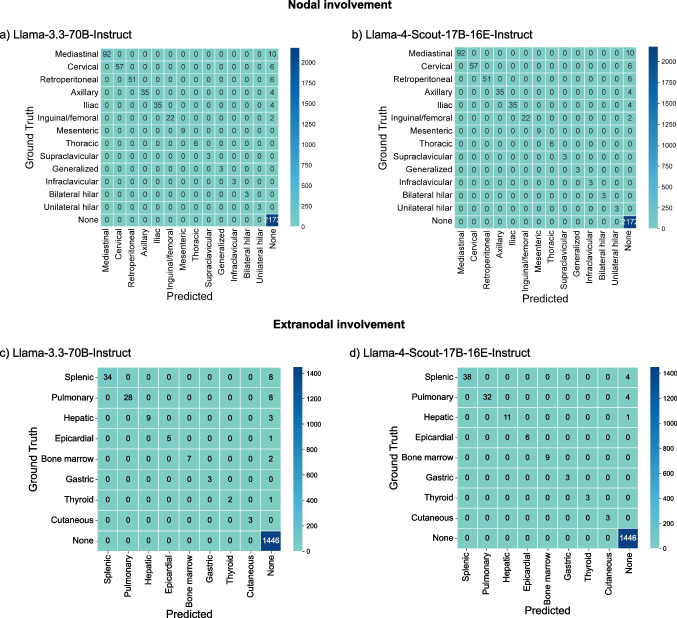
Confusion matrices that depict ground truth data with model predictions for nodal and extranodal involvement classification per site and model. For each matrix, the vertical axis represents the ground truth, and the horizontal axis shows the predictions: **a** Llama-3.3-70B-Instruct and **b** Llama-4-Scout-17B-16E-Instruct performance on the nodal involvement classification task, and **c** Llama-3.3-70B-Instruct and **d** Llama-4-Scout-17B-16E-Instruct on the extranodal involvement classification task

For the nodal extraction task, Llama-4-Scout-17B-16E-Instruct had an average accuracy of 0.99 (95% CI = 0.98–0.99), which was higher than Llama-3.3-70B-Instruct’s average accuracy of 0.97 (95% CI = 0.95–0.96; *p* = 0.001). For extranodal sites, Llama-4-Scout-17B-16E-Instruct outperformed Llama-3.3-70B-Instruct again, achieving an accuracy of 0.99 (95% CI = 0.99–1.00) versus 0.99 (95% CI = 0.98–0.99; *p* = 0.013). As neither model produced false-positive predictions, precision and specificity were 1.00 (95% CI = 1.00–1.00) for all nodal and extranodal analyses. Figure 2 shows class-wise confusion matrices for each model, comparing predicted labels with the ground truth. Complete performance metrics for each label and task are provided in Supplementary eTables 1 and 2.

#### Performance for Lugano Staging Classification/Treatment Response Classification

**Fig. 3:**
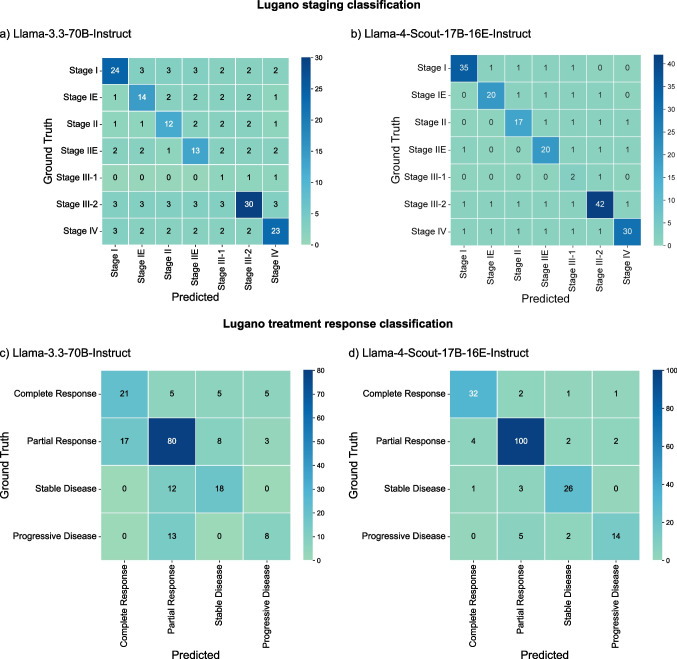
Confusion matrices that depict ground truth data with model predictions for Lugano staging and treatment response classification per class and model. For each matrix, the vertical axis represents the ground truth, and the horizontal axis shows the predictions: **a** Llama-3.3-70B-Instruct and **b** Llama-4-Scout-17B-16E-Instruct performance on the Lugano staging classification task, and **c** Llama-3.3-70B-Instruct and **d** Llama-4-Scout-17B-16E-Instruct on the Lugano treatment response classification task

For the prediction of the Lugano staging class, Llama-4-Scout-17B-16E-Instruct achieved an average accuracy of 0.85 (95% CI = 0.79–0.89), outperforming Llama-3.3-70B-Instruct, which reached 0.60 (95% CI = 0.53–0.67; *p* < 0.001). Because exactly one stage is predicted per patient, precision, recall, and F1 equal the reported accuracies (see Supplementary eTable 3). For the prediction of treatment response, Llama-4-Scout-17B-16E-Instruct again achieved a higher accuracy of 0.88 (95% CI = 0.83–0.92), whereas Llama-3.3-70B-Instruct achieved 0.65 (95% CI = 0.58–0.71; *p* < 0.001). The same values apply to precision, recall, and F1 (see Supplementary eTable 4). Figure 3 presents the class-wise confusion matrices for both models.

#### Error Analysis

Llama-4-Scout-17B-16E-Instruct produced a total of 64 errors and Llama-3.3-70B-Instruct 150 errors across all iterations and tasks (see Supplementary eTable 5). Relative to the total number of decisions per task, the highest error rate occurred in treatment response generation (20.5% of cases for Llama-3.3-70B versus 7.2% for Llama-4-Scout-17B), followed by the generation of Lugano staging (7.2% versus 4.6%). In the treatment response task, hallucinated residual disease (i.e., progressive disease (PD)/stable disease (SD) in cases with complete response (CR)) appeared in 5.1% of Llama-3.3-70B outputs and 1.5% of Scout-17B-16E outputs; missed residual disease (i.e., CR in cases with partial response (PR)) in 8.7% and 2.1%; and misjudged progression (i.e., PD in cases with PR or PD in cases with SD) in 6.7% and 3.6%, respectively. Under-staging errors were relatively infrequent with 1.0% of cases for both Llama-3.3-70B and Llama-4-Scout-17B, whereas over-staging occurred in 4.6% and 1.5%, and stage-confusion (e.g., II → IIE) in 1.5% and 2.1%, respectively. For extraction tasks involving nodal or extranodal disease, all errors were omissions of involved sites, with comparably low relative error rates (Llama-3.3-70B/Llama-4-Scout-17B: nodal, 2.9%/1.3%; extranodal, 1.5%/0.6%).

## Discussion

Our study demonstrates that the new Llama models can extract structured lymphoma disease data from radiology reports with high accuracy in a local, privacy-preserving hospital setting, adhering to the provided template structure in all cases. While both models were highly accurate in extracting nodal and extranodal disease sites, the newer Llama-4-Scout-17B-16E Instruct model significantly outperformed Llama-3.3-70B-Instruct. This performance gap widened for the reasoning tasks of assigning Lugano staging and treatment response classes, with Llama-4-Scout achieving 85%/88% accuracy compared to 60%/65% for Llama-3.3-70B-Instruct. Our systematic error analysis revealed that the highest relative error rates for both models occurred when interpreting the level of disease after treatment (specifically missing residual disease or misjudging progression), while for the generation of Lugano stages, incorrectly assigning a more advanced disease stage was the most common mistake. Notably, neither model produced hallucinations of newly involved nodal or extranodal sites. These findings suggest that while LLMs excel in extracting and structuring data, their performance declines when required to generate new clinical inferences. Our results are consistent with previous studies that have highlighted the potential of LLMs to transform unstructured radiology data into structured formats [13–21]. For instance, Adams et al. [15] demonstrated that GPT-4 achieved 100% accuracy in automatically aligning MRI and CT reports from various anatomical regions with their designated templates and structuring them without any errors or the introduction of extraneous findings. Similarly, open-source models such as Vicuna previously achieved high sensitivity and specificity for extracting key radiological features from brain MRI reports [27]. Similarly, our study demonstrates the strength of LLMs in handling clinical information retrieval tasks by highlighting the consistent and accurate extraction of patient lymphoma disease nodal and extranodal data. This capability holds significant potential for optimizing workflows in the evaluation and assessment of often lengthy oncologic disease processes, thereby improving time efficiency and reducing the burden on radiologists [28].

In contrast, the generation of complex clinical outputs based on radiological data, exemplified in our study by the assessment of the Lugano stage and treatment response, seems to remain a challenge for LLMs. These findings align with previous observations that, although LLMs are adept at extracting factual data, they struggle with tasks that require the integration of multifaceted clinical criteria and clinical reasoning [29, 30]. For example, assigning the correct Lugano stage requires not only the correct extraction of all nodal and extranodal sites but also the correct application of these findings to the staging criteria and their modifiers [24]. Similarly, assessing treatment response demands not only evaluating current disease involvement and staging criteria but also comparing it with previous radiological data to determine progression. Prompt engineering can play a critical role in reducing clinical errors of LLMs, and while we have provided the Lugano staging and treatment response criteria in the prompts as guidance and excluded modifiers for B-symptoms (which might not be available in the radiological data), there still remain multiple steps for the LLM to fail, including the incorrect extraction of disease involvement, the incorrect assessment of its spatial or temporal evolution, or the misinterpretation of the guidelines. Nevertheless, the CoT prompting strategy in our study achieved overall high performance, with the highest relative error rates found for hallucinated residual disease, missed residual disease, and misjudged progression. From a clinical point of view, prompting the model to explicitly list baseline findings, then post-treatment findings, and only then to compare them according to Lugano criteria makes its reasoning process also more transparent and allows for easier verification. Incorporating probabilistic scoring or self-critique steps could further down-weight borderline over-staging decisions and flag cases that require radiologist review [31]. For extraction omissions, incorporating retrieval-augmented generation (RAG) could be one solution, where LLMs retrieve information from dynamically updated knowledge bases [32, 33]. For example, a RAG system could first retrieve all document excerpts relevant to disease sites before passing them to the LLM for summarization, which may minimize the risk that relevant findings scattered throughout a long report are overlooked. Given that contemporary lymphoma guidelines, such as those from the European Society for Medical Oncology or the National Comprehensive Cancer Network, are regularly updated, RAG systems could also play an important role in keeping the model responses up-to-date [34, 35]. Without comprehensive, real-time linkage to such guideline repositories, however, LLM outputs risk becoming outdated or generic, potentially leading to suboptimal or incorrect clinical assessments [9].

Furthermore, research indicates that LLMs may struggle with preserving context over extended narratives, leading to potential inaccuracies in complex clinical documentation [36]. In our study, we employed Meta’s Llama-3.3-70B model, which features a context window of 128,000 tokens (i.e., around 96,000 words in English language), and compared it with the recently released Llama-4-Scout-17B-16E-Instruct, with a reported context window of up to 10,000,000 tokens [22]. The widening performance gap on reasoning tasks, whereas data extraction tasks were performed well by both models, suggests the interplay between clinical content complexity, the model’s reasoning capabilities, and the contextual dependencies of medical documentation, as described by Jin et al., may also play a role [37]. Thus, it appears that the capabilities of newer, even smaller LLMs may handle the cognitive demands of complex clinical scenarios involving multiple concepts, interdependencies, and precise terminology more effectively.

While we included two of the newest Llama models, open-source LLMs with reasoning capabilities, such as DeepSeek’s R1, which achieved performance comparable to OpenAI’s proprietary o1 on math, coding, and general reasoning benchmarks, may perform better on these clinical tasks [38]. However, their adoption in real-world clinical settings is limited by their need for computational resources, including high-performance graphics processing units, large storage capacities, and ongoing maintenance, all of which can strain hospital IT infrastructure [39]. On the other hand, while cloud-based solutions can reduce the need for local computing resources, they can also be costly. Cloud platforms such as Amazon Web Services charge based on usage, including model inference time, data storage, and bandwidth, making them expensive for continuous clinical use [40]. In addition, transferring sensitive patient data to cloud servers raises concerns about privacy, security, regulatory compliance (e.g., HIPAA, GDPR), and data sovereignty, adding another layer of complexity to their adoption in clinical environments [41]. Thus, the trade-off between the computational demands of local deployment and the financial and regulatory burdens of cloud-based solutions remains a critical barrier to the widespread clinical integration of advanced LLMs. Ultimately, while domain-specific fine-tuning of open-source LLMs was introduced with the goal to more effectively navigate medical documentation due to the intricate terminology and multifaceted interdependencies inherent in clinical data, recent evidence suggests that this does not lead to a performance increase [42]. Specifically, Dorfner et al. [43] showed that biomedically fine-tuned LLMs performed worse than general-purpose models on clinical tasks such as information extraction, document summarization, and clinical coding.

Our study has limitations. It is single-center and cross-sectional, based on data from a short period and adult lymphoma patients, which reduces its generalizability. Considering that about a quarter of the patients had stage III(2) disease and diffuse large B-cell lymphoma with an overall high prevalence of mediastinal involvement, the performance of LLMs in rarer subtypes remains an open question for future cohort studies. Additionally, we only evaluated two newer, open-source Llama models because of their cost-effectiveness and feasibility for local implementation. Therefore, future studies may evaluate and compare the use of different LLMs on real-world clinical data from more diverse patient populations using privacy-preserving approaches. We also did not explore alternative strategies such as few-shot learning or breaking down tasks into multiple, sequential prompts. A comparative analysis of different prompting techniques could yield further insights into optimizing LLM performance for this clinical application. In addition, future studies should investigate the incorporation of RAG-based approaches for data extraction and generation of disease progression reports, as well as the incorporation of multimodal data, such as imaging, laboratory values, and clinical history parameters, to further contextualize the feasibility of using LLMs in clinical decision-making.

## Conclusion

In conclusion, our study demonstrates that privacy-preserving, locally deployed LLMs can effectively extract and structure key clinical information from free-text lymphoma imaging reports. However, their moderate accuracy in generating complex inferences, such as the Lugano classification for staging and treatment response, highlights the need for further refinement of LLMs in the healthcare domain.

## Supplementary Information

Below is the link to the electronic supplementary material.

ESM 1 (DOCX 55.5 KB)

## Data Availability

Datasets generated during the current study are available from the corresponding author on reasonable request.

## Abbreviations

CI: Confidence interval
CR: Complete response
IQR: Interquartile range
GDPR: General data protection regulation
GPT: Generative pre-trained transformers
HIPAA: Health insurance portability and accountability act
LLM: Large language model
PD: Progressive disease
PR: Partial response
RAG: Retrieval-augmented generation
SD: Stable disease
