# Supplementary material for: Privacy-Preserving Generation of Structured Lymphoma Progression Reports from Cross-sectional Imaging: A Comparative Analysis of Llama 3.3 and Llama 4
Source: J Imaging Inform Med. 2025 Jul 25;39(2):1868–78. doi: 10.1007/s10278-025-01618-z (PMC13103223; doi:10.1007/s10278-025-01618-z)
Supplement: Supplementary file 1 — (DOCX 55.5 KB) [file 10278_2025_1618_MOESM1_ESM.docx]

**Supplementary Material**

**1. Chain-of-thought prompting strategy**

**Task A: Record the following key elements from the provided radiology reports:**

1. Note the lymphoma subtype
2. Note the date and type of examination
3. Nodal lymphoma involvement:
   1. Note all nodal sites (cervical, mediastinal, axillary, retroperitoneal, inguinal, etc.)
   2. Document size measurements if available
4. Extranodal lymphoma involvement:
   1. Note any organs, bone lesions, or other extralymphatic tissue involvement beyond lymph nodes
   2. Document size measurements if available

**Task B: Compare the most recent report with the previous report for the key elements from Task A**

1. **Order of Findings**

- Arrange all findings from earliest to most recent date.

1. **Identify Changes**

- For each nodal and extranodal site, compare sizes and descriptions across time points.

1. **Ensure Completeness & Accuracy**

- Verify that all locations mentioned in the source text appear in the compiled summary.
- Confirm that measurements, dates, and other details are consistent with the source reports.

**Task C: Determine the current Lugano stage using the extracted data from Task A and B according to the following rules:**

1. Stage I: One node or group of adjacent nodes.
2. Stage IE:** Single extralymphatic site in the absence of nodal involvement.
3. Stage II: Two or more nodal groups on the same side of the diaphragm.
4. Stage IIE: Contiguous extralymphatic extension from a nodal site with or without involvement of other lymph node regions on the same side of the diaphragm.
5. Stage III: Nodes on both sides of the diaphragm; OR nodes above the diaphragm with spleen involvement.
   1. Stage III(1): Spleen, hilar, celiac, or portal nodes involved.
   2. Stage III(2): Para-aortic, iliac, inguinal, or mesenteric nodes involved.
6. Stage IV: Diffuse or disseminated involvement of one or more extranodal organs (e.g., liver, bone marrow) beyond that designated 'E', with or without associated lymph node involvement.

**Task D: Determine the current Lugano treatment response class using the extracted data from Task A and B according to the following rules:**

1. Complete Response (CR):
   1. Nodal sites reduce to ≤1.5 cm in longest transverse diameter (LDi).
   2. Complete disappearance of all other radiologic evidence of disease.
2. Partial Response (PR):
   1. ≥50% decrease in the sum of the product of the perpendicular diameters (PPDs) of up to six representative lesions.
3. Stable Disease (SD):
   1. <50% decrease from baseline in the PPDs of up to six dominant, measurable lesions.
   2. No criteria for progressive disease are met.
4. Progressive Disease (PD):
5. New or Increased Adenopathy:
   1. An individual node is abnormal with:
      1. LDi >1.5 cm AND
      2. PPD increase by ≥50% from nadir (smallest recorded measurement) AND
      3. LDi or SDi (shortest axis) increases from nadir by >0.5 cm for lesions ≤2 cm, or >1.0 cm for lesions >2 cm.
6. Splenic Volume Increase:
   1. With prior splenomegaly: Increase in length by >50% of its prior increase beyond baseline.
   2. Without prior splenomegaly: Length increases by at least 2 cm.
   3. New or recurrent splenomegaly.
7. New or larger non-measured lesions.
8. Recurrence of previously resolved lesions.
9. A new extranodal lesion >1 cm in any axis.
10. A new node >1.5 cm in any axis.

**Task E: Compilation into a Template**

Use **Task A** (extraction), **Task B** (comparison), and **Task C/D** (Lugano staging and response classification determination) to populate the following template. The layout must be fully correct, with each section clearly labeled.

| **Section** | **Field** | **Content to Fill** |
| --- | --- | --- |
| **1. Examination History** | **Most recent cross-sectional imaging study** | [List examination date and type] |
|  | **Cross-sectional imaging study for comparison** | [List examination date and type] |
| **2. Lymphoma disease status** | **Lymphoma subtype** | [e.g., Classical Hodgkin, Follicular, etc.] |
|  | **Nodal involvement** | [List all nodal regions from the source, with measurements and chronological changes from previous to most recent, e.g., “Cervical node from 15 mm (22-03-2019) to 9 mm (29-09-2019)”] |
|  | **Extranodal involvement** | [List all extranodal sites, e.g., spleen, bone, or other tissues with size or lesion changes across time points] |
|  | **Lugano staging criteria** | [List the most recent disease stage according to Lugano] |
|  | **Lugano response criteria** | [List the most recent treatment response according to Lugano] |

**2. Tables**

**eTable 1. Performance metrics for nodal involvement by model and class.**

| **Nodal site** | **Recall** | **Precision** | **Specificity** | **F1-score** | **Accuracy** |
| --- | --- | --- | --- | --- | --- |
| Llama-3.3-70B-Instruct |  |  |  |  |  |
| Mediastinal lymph nodes | 0.80 | 1.00 | 1.00 | 0.89 | 0.90 |
| Cervical lymph nodes | 0.79 | 1.00 | 1.00 | 0.88 | 0.93 |
| Retroperitoneal lymph nodes | 0.81 | 1.00 | 1.00 | 0.89 | 0.94 |
| Axillary lymph nodes | 0.79 | 1.00 | 1.00 | 0.89 | 0.96 |
| Iliac lymph nodes | 0.82 | 1.00 | 1.00 | 0.90 | 0.96 |
| Inguinal/femoral lymph nodes | 0.79 | 1.00 | 1.00 | 0.88 | 0.97 |
| Mesenteric lymph nodes | 0.83 | 1.00 | 1.00 | 0.91 | 0.99 |
| Thoracic nodes (extra-mediastinal) | 0.78 | 1.00 | 1.00 | 0.88 | 0.99 |
| Supraclavicular lymph nodes | 0.67 | 1.00 | 1.00 | 0.80 | 0.99 |
| Generalized lymphadenopathy | 1.00 | 1.00 | 1.00 | 1.00 | 1.00 |
| Infraclavicular lymph nodes | 1.00 | 1.00 | 1.00 | 1.00 | 1.00 |
| Bilateral hilar lymph nodes | 0.67 | 1.00 | 1.00 | 0.80 | 0.99 |
| Unilateral hilar lymph nodes | 0.33 | 1.00 | 1.00 | 0.50 | 0.99 |
| Average (case-weighted), 95% CI | 0.80, 0.74-0.82 | 1.00, 1.00-1.00 | 1.00, 1.00-1.00 | 0.88, 0.85- 0.90 | 0.96, 0.95-0.96 |
| Llama-4-Scout-17B-16E-Instruct |  |  |  |  |  |
| Mediastinal lymph nodes | 0.90 | 1.00 | 1.00 | 0.95 | 0.95 |
| Cervical lymph nodes | 0.90 | 1.00 | 1.00 | 0.95 | 0.97 |
| Retroperitoneal lymph nodes | 0.89 | 1.00 | 1.00 | 0.94 | 0.97 |
| Axillary lymph nodes | 0.90 | 1.00 | 1.00 | 0.95 | 0.98 |
| Iliac lymph nodes | 0.90 | 1.00 | 1.00 | 0.95 | 0.98 |
| Inguinal/femoral lymph nodes | 0.92 | 1.00 | 1.00 | 0.96 | 0.99 |
| Mesenteric lymph nodes | 1.00 | 1.00 | 1.00 | 1.00 | 1.00 |
| Thoracic nodes (extra-mediastinal) | 1.00 | 1.00 | 1.00 | 1.00 | 1.00 |
| Supraclavicular lymph nodes | 1.00 | 1.00 | 1.00 | 1.00 | 1.00 |
| Generalized lymphadenopathy | 1.00 | 1.00 | 1.00 | 1.00 | 1.00 |
| Infraclavicular lymph nodes | 1.00 | 1.00 | 1.00 | 1.00 | 1.00 |
| Bilateral hilar lymph nodes | 1.00 | 1.00 | 1.00 | 1.00 | 1.00 |
| Unilateral hilar lymph nodes | 1.00 | 1.00 | 1.00 | 1.00 | 1.00 |
| Average (case-weighted), 95% CI | 0.91, 0.86-0.92 | 1.00, 1.00-1.00 | 1.00, 1.00-1.00 | 0.95, 0.92- 0.96 | 0.99, 0.98-0.99 |

Abbreviation: CI: confidence interval.

**eTable 2. Performance metrics for extranodal involvement by model and class.**

| **Extranodal site** | **Recall** | **Precision** | **Specificity** | **F1-score** | **Accuracy** |
| --- | --- | --- | --- | --- | --- |
| Llama-3.3-70B-Instruct |  |  |  |  |  |
| Splenic lesions | 0.81 | 1.00 | 1.00 | 0.90 | 0.95 |
| Pulmonary lesions | 0.78 | 1.00 | 1.00 | 0.88 | 0.96 |
| Hepatic lesions | 0.75 | 1.00 | 1.00 | 0.86 | 0.98 |
| Epicardial involvement | 0.83 | 1.00 | 1.00 | 0.91 | 0.99 |
| Bone marrow involvement | 0.78 | 1.00 | 1.00 | 0.88 | 0.99 |
| Gastric infiltration | 1.00 | 1.00 | 1.00 | 1.00 | 1.00 |
| Thyroid involvement | 0.67 | 1.00 | 1.00 | 0.80 | 0.99 |
| Cutaneous involvement | 1.00 | 1.00 | 1.00 | 1.00 | 1.00 |
| Average (case-weighted), 95% CI | 0.80, 0.72-0.86 | 1.00, 1.00-1.00 | 1.00, 1.00-1.00 | 0.89, 0.83- 0.93 | 0.99, 0.98- 0.99 |
| Llama-4-Scout-17B-16E-Instruct |  |  |  |  |  |
| Splenic lesions | 0.90 | 1.00 | 1.00 | 0.95 | 0.98 |
| Pulmonary lesions | 0.89 | 1.00 | 1.00 | 0.94 | 0.98 |
| Hepatic lesions | 0.92 | 1.00 | 1.00 | 0.96 | 0.99 |
| Epicardial involvement | 1.00 | 1.00 | 1.00 | 1.00 | 1.00 |
| Bone marrow involvement | 1.00 | 1.00 | 1.00 | 1.00 | 1.00 |
| Gastric infiltration | 1.00 | 1.00 | 1.00 | 1.00 | 1.00 |
| Thyroid involvement | 1.00 | 1.00 | 1.00 | 1.00 | 1.00 |
| Cutaneous involvement | 1.00 | 1.00 | 1.00 | 1.00 | 1.00 |
| Average (case-weighted), 95% CI | 0.92, 0.86-0.96 | 1.00, 1.00-1.00 | 1.00, 1.00-1.00 | 0.96, 0.92- 0.98 | 0.99, 0.99-1.00 |

Abbreviation: CI: confidence interval.

**eTable 3. Performance metrics for Lugano staging classification by model and class.**

| **Stage** | **Recall** | **Precision** | **Specificity** | **F1-score** | **Accuracy** |
| --- | --- | --- | --- | --- | --- |
| Llama-3.3-70B-Instruct |  |  |  |  |  |
| Stage I | 0.62 | 0.60 | 0.89 | 0.60 | 0.61 |
| Stage IE | 0.58 | 0.56 | 0.93 | 0.56 | 0.57 |
| Stage II | 0.57 | 0.52 | 0.93 | 0.52 | 0.54 |
| Stage IIE | 0.54 | 0.54 | 0.93 | 0.54 | 0.54 |
| Stage III-1 | 0.33 | 0.33 | 0.99 | 0.33 | 0.33 |
| Stage III-2 | 0.63 | 0.67 | 0.88 | 0.67 | 0.65 |
| Stage IV | 0.64 | 0.66 | 0.92 | 0.66 | 0.65 |
| Average (case-weighted), 95% CI | 0.60, 0.53-0.67 | 0.60, 0.53-0.67 | 0.93, 0.92-0.95 | 0.60, 0.53-0.67 | 0.60, 0.53-0.67 |
| Llama-4-Scout-17B-16E-Instruct |  |  |  |  |  |
| Stage I | 0.90 | 0.85 | 0.96 | 0.85 | 0.88 |
| Stage IE | 0.83 | 0.83 | 0.98 | 0.83 | 0.83 |
| Stage II | 0.81 | 0.77 | 0.97 | 0.77 | 0.79 |
| Stage IIE | 0.83 | 0.83 | 0.98 | 0.83 | 0.83 |
| Stage III-1 | 0.67 | 0.67 | 0.99 | 0.67 | 0.67 |
| Stage III-2 | 0.88 | 0.89 | 0.96 | 0.89 | 0.88 |
| Stage IV | 0.83 | 0.88 | 0.97 | 0.88 | 0.86 |
| Average (case-weighted), 95% CI | 0.85, 0.79- 0.89 | 0.85, 0.79- 0.89 | 0.98, 0.97- 0.98 | 0.85, 0.79- 0.89 | 0.85, 0.79- 0.89 |

Abbreviation: CI: confidence interval.

**eTable 4. Performance metrics for Lugano treatment response classification by model and class.**

| **Class** | **Recall** | **Precision** | **Specificity** | **F1-score** | **Accuracy** |
| --- | --- | --- | --- | --- | --- |
| Llama-3.3-70B-Instruct |  |  |  |  |  |
| Complete response | 0.58 | 0.55 | 0.89 | 0.57 | 0.84 |
| Partial response | 0.74 | 0.73 | 0.66 | 0.73 | 0.70 |
| Stable disease | 0.60 | 0.58 | 0.92 | 0.59 | 0.87 |
| Progressive disease | 0.38 | 0.50 | 0.95 | 0.43 | 0.89 |
| Average (case-weighted), 95% CI | 0.65, 0.58-0.71 | 0.65, 0.58-0.71 | 0.93, 0.92- 0.95 | 0.65, 0.58-0.71 | 0.65, 0.58-0.71 |
| Llama-4-Scout-17B-16E-Instruct |  |  |  |  |  |
| Complete response | 0.89 | 0.86 | 0.97 | 0.88 | 0.95 |
| Partial response | 0.93 | 0.91 | 0.89 | 0.92 | 0.91 |
| Stable disease | 0.87 | 0.84 | 0.97 | 0.85 | 0.95 |
| Progressive disease | 0.67 | 0.82 | 0.98 | 0.74 | 0.95 |
| Average (case-weighted), 95% CI | 0.88, 0.83-0.92 | 0.88, 0.83-0.92 | 0.88, 0.83-0.92 | 0.88, 0.83-0.92 | 0.88, 0.83-0.92 |

Abbreviation: CI: confidence interval.

**eTable 5. Error analysis by model and task.**

| **Error type** | **Error description** | **Llama-3.3-70B-Instruct, total n (%)** | **Llama-4-Scout-17B-16E-Instruct, total n (%)** | **Example excerpt original report** | **Example excerpt generated template** |
| --- | --- | --- | --- | --- | --- |
| Nodal involvement | | | | | |
| Incomplete | Missing nodal sites | 73 (2.9%) | 32 (1.3%) | Significant decrease in the size of the large lymphoma mass in the mediastinum, currently measuring a maximum of 5.7 x 8.8 cm (previously 13 x 12 cm). […] The circumferential tumor bulk along the mesenteric root with aortic encasement has also markedly decreased in size, now with a maximum measurement of 2.1 cm in the dorsoventral dimension (previously 3.9 cm). […] The right inguinal lymph node has decreased in size, with a current maximum axial short-axis diameter of 1.1 cm (previously 2.3 cm). | - Mediastinal lymphoma mass from 13 x 12 cm (22-06-2023) to 5.7 x 8.8 cm (24-09-2023)  - Right inguinal lymph node from 2.3 cm (22-06-2023) to 1.1 cm (24-09-2023)  Error interpretation: The tumor bulk along the mesenteric root was likely not recognized as a nodal site. |
| Extranodal involvement | | | | | |
| Incomplete | Missing extranodal sites | 23 (1.5%) | 9 (0.6%) | The spleen is mildly decreased in size on coronal images, measuring 11.4 cm in maximum diameter (previously 12.0 cm). A 1.3 cm splenic lesion (slice position: 448.6) demonstrates an iris-diaphragm phenomenon, unchanged in size and attenuation. An additional 1.1 cm contrast-enhancing lesion (slice position: 457.6) is also stable from the previous study. The remaining focal splenic lesions, both hypo- and hyperdense, are currently not well delineated. | - Spleen size from 12 cm (08-12-2023) to 11.4 cm (15-03-2024)  - Stable splenic lesions measuring 1.3 and 1.1 cm  Error interpretation: Further focal splenic lesions that are both hypo- and hyperdense are omitted, likely because they are described as not well-delineated in the original report. |
| Lugano staging classification | | | | | |
| Under-staging (e.g., Stage IV -> III) | Missing disseminated disease or nodal stations | 2 (1.0 %) | 2 (1.0 %) | Small hepatic lesions in segments VII and VI, largest measuring 1.8 x 1.4 cm. [...] Residual mediastinal lymphadenopathy with largest node 2.8 x 2.1 cm. Multiple enlarged retroperitoneal nodes, largest para-aortic node 3.2 x 2.4 cm. […] Bilateral iliac adenopathy. […] The spleen is normal in size, with a single 8 mm low-density focus in the splenic interpolar region. | Lugano Staging Classification: Stage: IV  Error interpretation: Correct stage is IV. The hepatic lesions may have been misinterpreted as not being lymphoma related. However, they represent disseminated involvement of an extralymphatic organ. |
| Over-staging (e.g., Stage I -> II) | Hallucinated disease; misinterpreting benign nodes | 9 (4.6 %) | 3 (1.5 %) | Single enlarged left cervical lymph node measuring 3.4 x 2.8 cm. […] Multiple bilateral axillary lymph nodes measuring 6-8 mm. | Lugano Staging Classification: Stage: II  Error interpretation: Correct stage is I due to involvement of only a single lymph node region. Normal-sized bilateral axillary nodes do not represent lymphadenopathy. |
| Confusion (e.g., II -> IIE) | Misinterpretation of extranodal extension rules | 3 (1.5 %) | 4 (2.1 %) | Residual right hilar mass measuring 3.2 x 2.4 cm with extension into right upper lobe and poorly defined margins. […] Right paratracheal nodes with size decrease, largest now 1.6 cm. | Lugano Staging Classification: Stage: II  Error interpretation: Correct stage is IIE due to the contiguous extralymphatic extension into the right upper lobe. |
| Lugano treatment response classification | | | | | |
| Hallucinated residual disease (CR -> PR/SD) | Misinterpreting post-treatment changes | 10 (5.1 %) | 3 (1.5 %) | Left cervical lymph node with a reduction in size of 1.2 cm (previously 4.5 cm). […] Previously described right paratracheal nodes can no longer be delineated. | Lugano Treatment Response  Classification: [x] Partial Response  Error interpretation: Correct is complete response. Single cervical node has reduced to ≤1.5 cm in longest transverse diameter, paratracheal nodes have completely resolved. |
| Missing residual disease (PR -> CR) | Failure to capture remaining active tumor | 17 (8.7 %) | 4 (2.1 %) | Left cervical conglomerate reduced from a short-axis diameter of 2.3 to 1.2 cm. […] Persistent mesenterial nodes, exemplary at slice position 319.2. | Lugano Treatment Response  Classification: [x] Complete Response  Error interpretation: Correct is partial response due to persistent mesenterial nodes. |
| Misjudged progression (PR  -> PD or SD -> PD) | Misinterpretation of prior scan; over-sensitivity to minor changes | 13 (6.7 %) | 7 (3.6 %) | Mediastinal adenopathy stable with largest node measuring 2.1 x 1.6 cm. […] Para-aortic nodes show increase in size, the largest now 2.0 x 1.4 cm compared to 1.6 x 1.2 cm on prior study. | Lugano Treatment Response  Classification: [x] Progressive Disease  Error interpretation: Correct is stable disease. While para-aortic nodes show PPD increase by ≥50%, the diameter did not increase by >0.5 cm from nadir as it would be required for lesions ≤2 cm. |

Abbreviations: CR: complete response; PR: partial response; PD: progressive disease; PPD: perpendicular diameter; SD: stable disease.

Notes: Percentages are calculated against the total number of model decisions for each task (n=2535 nodal, n=1560 extranodal, n=195 Lugano staging classification, n=195 Lugano treatment response classification).
